# The Effects of Chinese Dwarf Cherry (*Cerasus humilis*) Kernel Oil on Defecation and the Gut Microbiota in Constipated Mice

**DOI:** 10.3390/nu18020319

**Published:** 2026-01-19

**Authors:** Jingyu Gao, Yumin Dai, Zhe Liang, Nan Chen, Xilong Li, Xin Wen, Yuanying Ni, Mo Li

**Affiliations:** 1 College of Food Science and Nutritional Engineering, China Agricultural University, No. 17 Qinghua East Road, Beijing 100083, China; b20223060512@cau.edu.cn (J.G.); 17801035462@163.com (Y.D.); liangzhe@cau.edu.cn (Z.L.); 18801086980@163.com (N.C.); lixilong2026@outlook.com (X.L.); xin.wen@cau.edu.cn (X.W.); nyy@cau.edu.cn (Y.N.); 2 National Engineering Research Center for Fruit and Vegetable Processing, No. 17 Qinghua East Road, Beijing 100083, China

**Keywords:** Chinese dwarf cherry (*Cerasus humilis)*, kernel oil, nutritive compound, constipation, gut microbiota

## Abstract

Background: The Chinese dwarf cherry (CDC) has been valued for over 2000 years for its medicinal and nutritional properties, particularly its kernels. Despite its recognition as a rich source of oil, the potential health benefits of CDC kernel oil remain unclear. Method: Initially, we evaluated the preventive effectiveness of CDC in a mouse model of constipation induced by loperamide. Results: The findings indicated that CDC kernel oil alleviated constipation by reducing the first black fecal defecation time and increasing the fecal number, wet weight, water content and gastrointestinal transit rate in model mice. Additionally, CDC kernel oil reduced inhibitory neurotransmitters and increased excitability neurotransmitters, two anti-oxidases’ activity and fecal short-chain fatty acid (SCFA) content. Histological analysis revealed an improved mucus cell morphology in the intestinal tract. Furthermore, CDC kernel oil increased the abundance of some beneficial bacteria. It was identified that the gut microbiota was associated with neurotransmitters, mediators of inflammation and SCFAs. Conclusion: The findings offer a scientific foundation for considering CDC kernel oil as a potential functional food for the alleviation of constipation.

## 1. Introduction

Constipation is a widespread and persistent issue globally, manifesting through symptoms like excessive effort during bowel movements, firm feces and rare occurrences of defecation [1]. The primary causes of chronic constipation can be categorized into three main areas: lifestyle-related factors, constipation-predominant irritable bowel syndrome and defecatory dysfunction [2]. Functional constipation is prevalent in 95% of individuals, with chronic constipation and irritable bowel syndrome with constipation constituting the two primary manifestations of this condition [1]. Constipation exerts considerable clinical, economic and quality of life impacts and is associated with notably elevated levels of psychological distress [3]. However, the mechanisms leading to constipation remain unclear. Some research discovered that the levels of neurotransmitters that regulate the enteric nervous system and gastrointestinal peristalsis were changed in patients suffering from constipation [4]. Furthermore, potential pathophysiological mechanism underlying constipation may involve alterations in aquaporin expression, disturbing the colon’s water transport mechanism. Recently, it has been found that constipation is also related to the gastrointestinal microbiota, which might influence bowel habits, gastrointestinal motility and stool consistency [5].

Functional constipation can be managed with a range of effective pharmacological treatments, including stimulant laxatives, stool softeners, etc. Nonetheless, these treatments do not always provide adequate relief. Moreover, excessive use of these therapies can lead to adverse effects such as electrolyte disturbances and melanosis coli. Consequently, it would be useful to develop more efficient and safe treatments for functional constipation. Some nutraceuticals have been discovered to aid in preventing or treating constipation, including senna, frangula, aloe, cascara and rhubarb [6]. It was reported that they promote defecation by reversing altered motility patterns and enhancing the colonic fluid volume, thereby facilitating the process [7]. Additionally, some researchers have also reported that plant oils can aid in the alleviation of constipation symptoms in children [8].

Chinese dwarf cherry (*Cerasus humilis*, CDC), a widely cultivated fruit crop in Northern China, can be consumed as a fresh fruit. It is also further processed into various derivative products, including canned or frozen cherries, wines, juices and jams. During these processing operations, kernels are generated as by-products, which are often discarded, despite their potential nutritional and medicinal value. Historically, the kernel of CDC was known as Yu Li Ren, and it has been utilized for both dietary and therapeutic purposes for over two millennia [9]. The CDC kernel boasts a wealth of nutrients, encompassing oils, proteins and fats, as well as an array of amino acids and minerals. Therefore, it exhibits a diverse range of physiological actions that have proven beneficial for individuals with heart disease and hypertension. Mu et al. [10] reported that these kernels are a rich source of oil (34–50%, *w*/*w*), and the oil extracted from the kernels exhibits a favorable fatty acid profile, including unsaturated fatty acids such as oleic and linoleic acid, which might contribute to its nutritional and functional benefits, such as antioxidant and anti-inflammatory effects. However, regarding research on CDC, although some topics have been explored, the study of the kernel oil’s bioactive functions stands out as an area that has received little attention. Given the growing emphasis on sustainable resource utilization, and the unique physicochemical properties and bioactive components of CDC kernel oil, the valorization of cherry processing by-products, namely the oil-rich kernels, presents a promising avenue toward enhancing food security and reducing agricultural waste.

This study investigated the effects of CDC kernel oil in alleviating loperamide-induced constipation in mice, while assessing alterations in fecal characteristics, particularly the first black fecal defection time, fecal number, wet weight, water content and colonic biochemical index, including nitric oxide (NO), malondialdehyde (MDA), glutathione peroxidase (GHX-PX) and superoxide dismutase (SOD), and the plasma biochemical index, including motilin (MTL), gastrin (GAS), somatostatin (SS), endothelin (ET), substance P (SP), vasoactive intestinal peptide (VIP), interleukin-6 (IL-6), interferon-α (IFN-α), interferon-γ (IFN-γ) and tumor necrosis factor-α (TNF-α). Moreover, we conducted measurements to assess alterations in the fecal microbiota composition, with the aim of elucidating the mechanisms by which exposure to CDC kernel oil affects gastrointestinal function. Through examining physiological responses to CDC kernel oil in models, we aimed to scientifically substantiate its potential therapeutic use for gastrointestinal motility disorders.

## 2. Materials and Methods

### 2.1. Materials

Chinese dwarf cherry (CDC, Jing’ou No. 1), planted in Inner Mongolia, was purchased from Shanxi Yunqi Biological Technology Co., Ltd. (Taiyuan, Shanxi, China). Kunming (KM) mice, aged 6 weeks old, were purchased from Charles River Laboratory Animal Technology Co., Ltd. (Beijing, China). All animal experiments adhered to the guidelines outlined in Animal Research: Reporting In Vivo Experiments (ARRIVE) 2.0.

### 2.2. Extraction of Oil from CDC Kernels

CDC kernels underwent drying to achieve a constant weight at 60 °C within a forced-air drying cabinet (DHG-9053a, Jinghong Experimental Equipment Co., Ltd., Shanghai, China). Subsequently, they were milled into a fine power using an electric mill and sieved manually through a mesh with a size of 600 μm. Extraction of the total oil content from the kernels was carried out through the use of supercritical carbon dioxide fluid extraction. Through a preliminary experiment conducted to optimize the extraction process, the extraction conditions were as follows: 500 g of the kernel flour, extraction time 1 h, extraction temperature 45 °C, extraction pressure 25 Mpa, separating pressure 5 Mpa, separating temperature 35 °C. The sterol compositions in CDC kernels oil were determined by GC-MS [11]. All of the nutrient analysis of CDC kernels oil was shown in Supplementary Materials (Sections 1.1 and 2.1).

### 2.3. Animal Experiments

#### 2.3.1. Animal Model Construction and Diet Treatment

Prior to the experimental protocols, the animals were kept in the following room conditions: relative humidity 40 ± 10%, a temperature of 25 ± 2 °C and a 12 h light/12 h dark cycle. The mice had unrestricted access to standard laboratory feed and distilled water. The commencement of all animal experiments followed a minimum acclimation period of one week.

**Figure 1:**
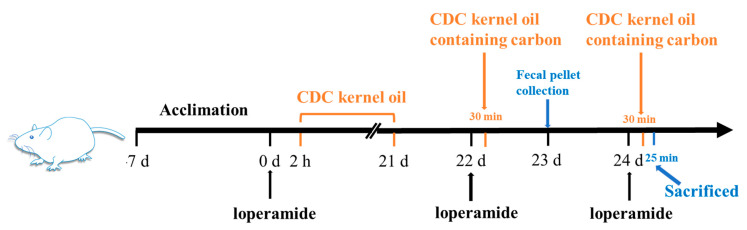
Animal experimentation process.

Following a seven-day adaptation period, 50 mice were randomly assigned to 5 groups, with 10 mice in each group, and labeled the control group, model group, low-dose oil group (LDO group, 1 g/kg), medium-dose oil group (MDO group, 2 g/kg) and high-dose oil group (HDO group, 3 g/kg). Initially, the mice were administered loperamide for 2 h, except for the control group, which was administered sterile saline. After this, the mice were intragastrically administered CDC kernel oil (LDO, MDO and HDO groups) or sterile saline (control and model groups) for 3 weeks at a volume of 100 μL/10 g·bw once a day. The mice were weighed weekly. The animal experimentation process is shown in Figure 1.

The sample size per group (*n* = 10) was initially chosen based on common practice in comparable studies using the same model and primary endpoints [1,12,13]. To confirm the adequacy of this sample size, a post hoc power analysis was performed upon study completion using the actual experimental data. For the primary comparison (first black fecal defecation time and gastrointestinal transit rate) between the model group and CDC kernel oil groups, the observed effect size (Cohen’s d) was calculated. Using this effect size, with α = 0.05 and the actual sample size per group (*n* = 10), the achieved power was >0.99 (G*Power software, version 3.1.9.7, Gachenbach, Germany), indicating a high likelihood of detecting the observed effect.

#### 2.3.2. Determination of Initial Bowel Movement Time, Number of Fecal Pellets and Fecal Water Content

After treatment for 3 weeks, the mice underwent an overnight fast (12 h), with access to water maintained throughout. On day 22, the model group and LDO, MDO and HDO groups received the intragastric administration of 100 μL/10 g·bw of loperamide (10 mg/kg), and the control group received the intragastric administration of 100 μL/10 g·bw of sterile saline. After 30 min, the control group and model group were given carbon, and the LDO, MDO and HDO groups were given CDC kernel oil containing activated carbon by intragastric administration at a volume of 100 μL/10 g·bw. The feces were collected over 6 h and were dried at 65 °C to a constant weight. Subsequently, the feces weight was documented. Furthermore, the defecation time of the first feces, the defecation weight and the number of fecal particles were recorded. The calculation of the fecal water content was performed as follows: water content of feces (%) = [dried weight of feces (g)]/[initial weight of feces (g)] × 100%. On day 23, sterile cryopreservation tubes were used to collect the feces and stored at −80 °C for further experiments. Subsequently, the mice underwent an overnight fast (12 h) with continued access to water [14]. The animal experimentation process is shown in Figure 1.

#### 2.3.3. Determination of Intestinal Propulsion Rate

On day 24, the model, LDO, MDO and HDO groups received the intragastric administration of 100 μL/10 g·bw of loperamide (5 mg/kg) and the control group received the intragastric administration of 100 μL/10 g·bw of sterile saline. After 30 min, the LDO, MDO and HDO groups received the intragastric administration of 100 μL/10 g·bw of CDC kernel oil containing activated carbon, and the control group and model group received the intragastric administration of sterile saline containing activated carbon. After 25 min, all mice were anesthetized, and blood samples from the retro-orbital venous sinus were obtained. Additionally, the entire intestinal tract was excised. The intestinal segment was measured for its length. The distance traveled by the activated carbon from the pylorus to the leading edge of the carbon bolus was determined. Using a specific formula, the gastrointestinal transport rate (GI) was subsequently calculated: gastrointestinal transit = migration distance of activated carbon/total intestine length × 100% [14].

### 2.4. Sample Collection and Measurement

Immediately following anesthesia, blood samples were collected from the mice via ocular extraction. After adding an anticoagulant and standing for 0.5 h, the collected blood was centrifuged at 3500 *g* for 15 min at 4 °C. The serum levels of MTL, GAS, SS, ET, VIP, SP, TNF-α, IFN-α, IFN-γ and IL-6 were measured using ELISA kits (Beijing Huaying Institute of Biotechnology, Beijing, China) [12].

For further analysis, colon tissue was collected and mixed with 0.85% saline at a ratio of 1:2 (g/mL) and was ground in an ice-water bath. Subsequently, the supernatant was collected after centrifuging at 3500 rpm for 15 min at 4 °C, and NO, MDA, GHX-PX, SOD and T-AOC were determined. Sections of colon tissue, measuring 2.0 cm, and the small intestine, located 2 cm from the cecum and also measuring 2.0 cm, were promptly clipped and fixed in formalin. Both colon and small intestine samples were embedded in paraffin, sliced into sections ranging from 3 to 6 μm in thickness and stained with hematoxylin and eosin. Subsequently, these sections were imaged using a light microscope equipped with a camera [15].

### 2.5. SCFA Concentrations Quantified by GC-MS

The SCFAs in the feces were analyzed by GC-MS (Agilent 7890B/5977A). Here, 100 mg of feces was taken into a 2 mL grinding tube with a steel ball. A mixture of 900 μL methanol and 100 μL 2-ethylbutyric acid (1000 g/mL), serving as an internal standard, was introduced, followed by two grinding procedures. After ultrasound in an ice-water bath and centrifuging, the supernatant was collected and mixed with anhydrous sodium sulfate. The mixture was again centrifuged and analyzed by GC-MS. The standard solutions of SCFAs were 5, 50, 100, 200, 300, 400 and 500 μg/mL with 2-ethylbutyric acid at 100 μg/mL [16].

### 2.6. DNA Extraction, Sequencing and Bioinformatics Analysis

DNA was isolated from fecal samples using a specialized kit (Omega Bio-Tek, Norcross, GA, USA) and its quantity/purity verified with a NanoDrop2000 spectrophotometer (Thermo Fischer Scientific, Waltham, MA, USA). PCR amplification of the bacterial 16S rRNA V3-V4 region was conducted, followed by the purification and quantification of the PCR products. These were then sequenced (2 × 300) on an Illumina MiSeq platform (Illumina, San Diego, CA, USA). Operational taxonomic unit (OTU) picking was performed using the Usearch software (version 7.1) with ≥97% sequence similarity, and microbial community analysis was performed [17].

### 2.7. Statistical Analysis

The experiments were performed three times, and the collected data underwent evaluations for normality and variance homogeneity. For data meeting these criteria, one-way analysis of variance (ANOVA, SPSS 13.0, IBM Corporation, Armonk, NY, USA) and Dunnett’s test were employed. All results were expressed as the mean ± standard. A statistically significant difference was indicated between the two sample groups when *p* < 0.05.

## 3. Results

### 3.1. Effects of CDC Kernel Oil on Fecal Parameters and Gastrointestinal Transit Rate

Prior to the formal in vivo study, we conducted a preliminary animal experiment to assess the toxicity of CDC kernel oil. The maximum dose of 15 g/kg∙bw of CDC kernel oil was used to gavage mice, all the mice survived and did not show symptoms of poisoning, so it can be preliminarily determined that CDC kernel oil is non-toxic (Supplementary Materials, Table S2). 

**Figure 2:**
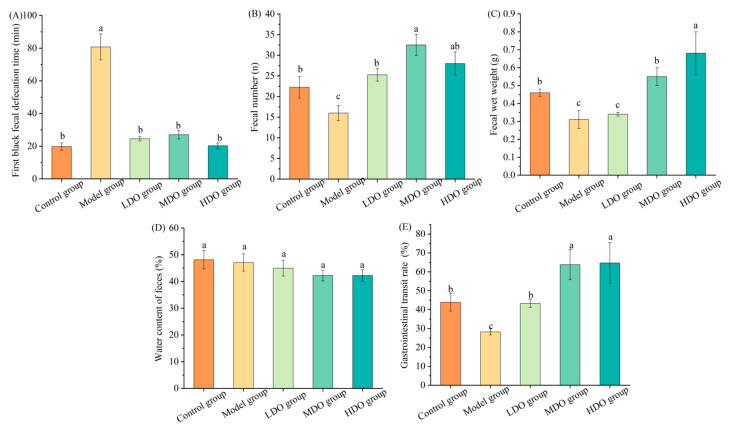
The impact of Chinese dwarf cherry kernel oil on fecal properties in different mouse groups (LDO group: low-dose oil group, MDO group: medium-dose oil group, HDO group: high-dose oil group). (**A**), first black fecal defecation time; (**B**), fecal number; (**C**), Fecal water weight; (**D**), water content of feces; (**E**), gastrointestinal transit rate. Note: The error bars represent the standard deviation (SD). Different lowercase letters indicate significant differences between different groups for the same indicator (*p* < 0.05).

No notable differences were observed in body weight or food consumption across the different mouse groups in the formal in vivo study. The data presented in Figure 2A–E reveal that, compared with the control group, the model group demonstrated a delayed first black fecal excretion time, alongside notable reductions in fecal count, fecal weight and gastrointestinal transit rate (all *p* < 0.05), suggesting constipation induction by loperamide in these mice. Subsequent CDC kernel oil administration in the LDO, MDO and HDO groups led to a shortened first black fecal excretion time compared to the model group (*p* < 0.05). Moreover, the fecal count in the LDO and HDO groups increased relative to the model group (*p* < 0.05), aligning closely with the control group (*p* > 0.05). Regarding the feces weight, both the MDO and HDO groups exhibited higher weights than the model group (*p* < 0.05). In terms of gastrointestinal transit rates, the LDO group showed an improvement compared to the model group (*p* < 0.05). Furthermore, the MDO and HDO groups demonstrated higher gastrointestinal transit rates than both the control and model groups (*p* < 0.05).

### 3.2. Effects of CDC Kernel Oil on Colonic Biochemical Indices of Mice

**Figure 3:**
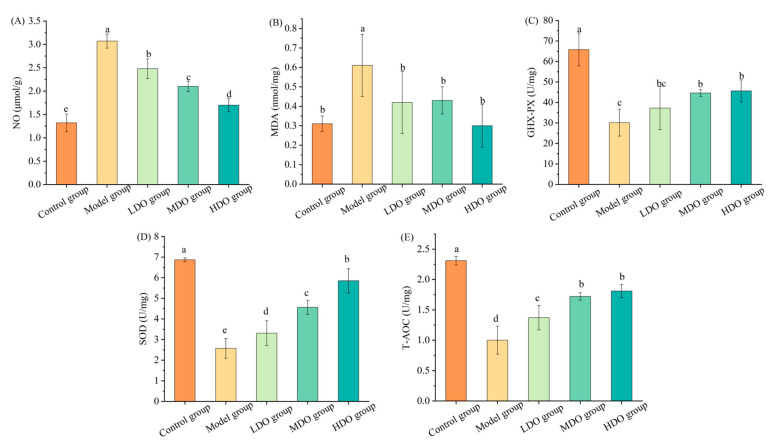
The impacts of Chinese dwarf cherry kernel oil on colonic biochemical indices in different mouse groups (LDO group: low-dose oil group, MDO group: medium-dose oil group, HDO group: high-dose oil group). (**A**), NO: nitric oxide; (**B**), MDA: malondialdehyde; (**C**) GHX-PX: glutathione peroxidase; (**D**) SOD: superoxide dismutase; (**E**) T-AOC: total antioxidant capacity. Note: The error bars represent the standard deviation (SD). Different lowercase letters indicate significant differences between different groups for the same indicator (*p* < 0.05).

In the colon tissue of the LHI group, there was a notable 2.33-fold increase in the inhibitory neurotransmitter NO compared to the control group (Figure 3A, *p* < 0.05). The administration of CDC kernel oil markedly mitigated this elevation in NO levels, particularly in the HDO group, which exhibited a 44.63% reduction compared to the model group. Concurrent with the increased NO production, there was the overproduction of MDA due to an upsurge in free radicals. Specifically, the MDA content in the model group was notably elevated when compared to the control group (*p* < 0.05, Figure 3B). Nevertheless, following CDC kernel oil treatment, the MDA content was reduced significantly compared with the model group (*p* < 0.05). GSH-PX and SOD, two essential anti-oxidases in the body, are pivotal in mitigating the detrimental effects of hydrogen peroxide and maintaining the oxidation–antioxidant balance in humans, as noted by Zhu et al. [18]. In vivo, NO can decompose into hydroxyl free radicals and nitrogen dioxide free radicals, inhibit the synthesis of the two enzymes, damage the molecular structures of the two enzymes and produce strong toxic effects on the body. The activity of GSH-PX and SOD in the model group was notably lower than that in the control group (Figure 3C,D *p* < 0.05). Both the MDO and HDO groups exhibited significant improvements in GSH-PX activity compared to the model group (*p* < 0.05). As for SOD activity, it showed a gradual increase with a higher dose of CDC kernel oil, resulting in significantly elevated levels compared to the model group (*p* < 0.05). T-AOC, a key indicator of the body’s total antioxidant capacity [19], was also found to be lower in the colon tissue of the model group compared to the control group (*p* < 0.05, Figure 3E). In contrast, the T-AOC values of the LDO, MDO and HDO groups were significantly improved compared to the model group (*p* < 0.05). These results suggest that CDC kernel oil can mitigate the oxidative effects induced by loperamide in colon tissue.

### 3.3. Effects of CDC Kernel Oil on Plasma Biochemical and Inflammatory Indices of Mice

**Figure 4:**
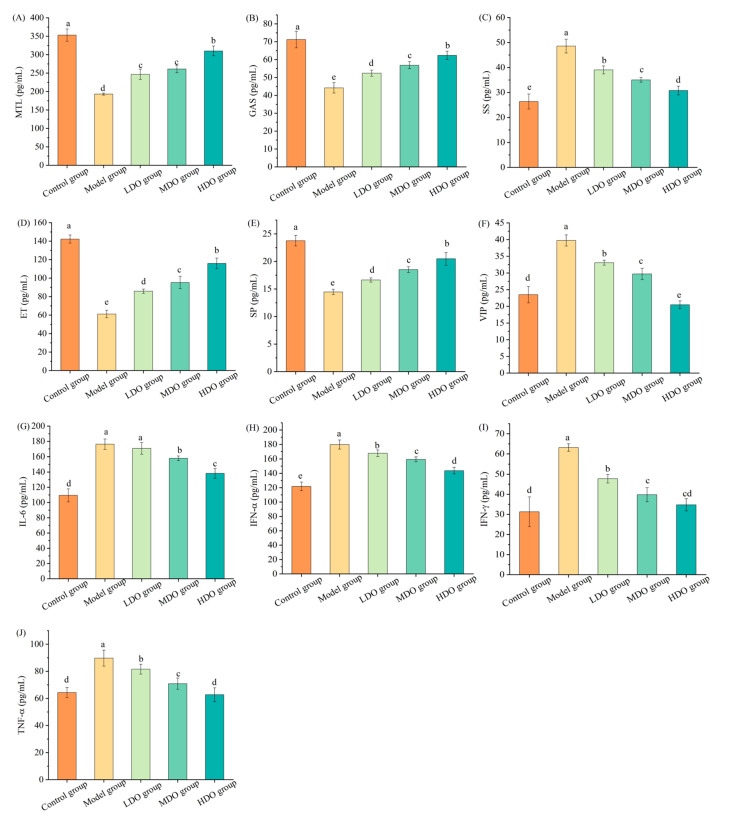
The impacts of Chinese dwarf cherry kernel oil on plasma biochemical indices in different mouse groups (LDO group: low-dose oil group, MDO group: medium-dose oil group, HDO group: high-dose oil group). (**A**) MTL: motilin; (**B**) GAS: gastrin; (**C**) SS: somatostatin; (**D**) ET: endothelin; (**E**) SP: substance P; (**F**) VIP: vasoactive intestinal peptide; (**G**) IL-6: interleukin-6; (**H**) INF-α: interferon-α; (**I**) INF-γ: interferon-γ; (**J**) TNF-α: tumor necrosis factor-α. Note: The error bars represent the standard deviation (SD). Different lowercase letters indicate significant differences between different groups for the same indicator (*p* < 0.05).

The data presented in Figure 4A–F reveal that the model group exhibited decreases in plasma MTL, GAS, ET and SP (*p* < 0.05) compared with the control group, whereas the SS and VIP levels were elevated (*p* < 0.05). Conversely, in the LDO, MDO and HDO groups, the concentrations of MTL, GAS, ET and SP were notably higher than in the model group (*p* < 0.05), with the SS and VIP levels being markedly lower (*p* < 0.05). The influence of CDC kernel oil displayed a dose-dependent trend, with the HDO group demonstrating the most pronounced effects.

As shown in Figure 4G–J, the plasma IL-6, IFN-α, IFN-γ and TNF-α levels in the model group were all higher than in the control group (*p* < 0.05), increased by 61.12%, 47.59%, 101.73% and 39.56%, respectively. After CDC kernel oil treatment, the inflammatory factor levels significantly decreased (*p* < 0.05) with an increase in the CDC kernel oil dose. These results suggest that CDC kernel oil ameliorates the inflammation caused by constipation.

### 3.4. Effects of CDC Kernel Oil on the Structure of the Small Intestine and Colon Tissue

**Figure 5:**
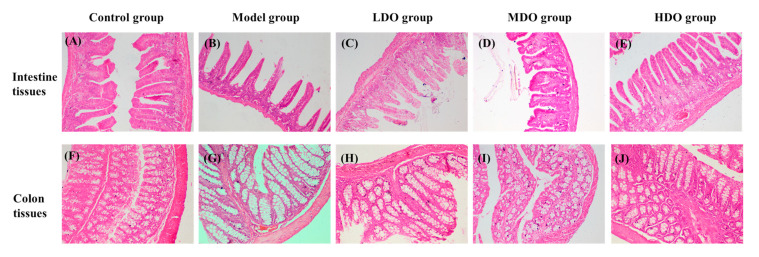
The impacts of Chinese dwarf cherry kernel oil on the small intestine tissue (**A**–**E**) and colon tissue (**F**–**J**) of mice (×200 magnification).

The results of the microscopic examination of the small intestine and colon tissue are illustrated in Figure 5A–J and Table 1. It was observed that the small intestinal villi in the control group (Figure 5A) were closely aligned and arranged with a uniform length. In contrast, we observed relatively scattered and disorganized small intestinal villi arrangements with damage in the model group (Figure 5B). The length of the small intestinal villi was decreased by 55.55%. After CDC kernel oil treatment, the length of the villi in the LDO group (Figure 5C) was increased by 33.31% compared with the LHI group. The villus density in the MDO group (Figure 5D) was recovered, with a uniform length and orderly arrangement, and the length of the villi was increased by 71.33%. In the HDO group (Figure 5E), we observed similarly arranged small intestinal villi to the control group, with no obvious damage. This indicates that CDC kernel oil has the capacity to notably mitigate the intestinal villi damage caused by constipation, with a more pronounced remedial effect observed at higher doses (*p* < 0.05).

As seen in Figure 5F–J and Table 1, the control group (Figure 5F) exhibited secure attachment between the mucosal muscle layer and the colon submucosa, with cells arranged in an orderly fashion. The colonic villi were neatly folded and firmly adhered to the mucosa. In contrast, the model group (Figure 5G) displayed minor inflammatory cell infiltration in colon tissue, accompanied by a scarcity of goblet cells and shallower crypts (decreased by 60.35%) compared to the control group. The administration of CDC kernel oil (Figure 5H–J) alleviated the damage to the colon tissue caused by loperamide, particularly in the HDO group (increased by 95.61% compared to model group) (Figure 5J). Here, the muscularis, submucosa and mucosa layers were distinctly visible, alongside well-defined crypts, goblet cells and large intestine glands. Notably, the colon tissue of the HDO group exhibited minimal pathological alterations.

### 3.5. Effects of CDC Kernel Oil on Short-Chain Fatty Acids (SCFAs) in Feces of Mice

**Figure 6:**
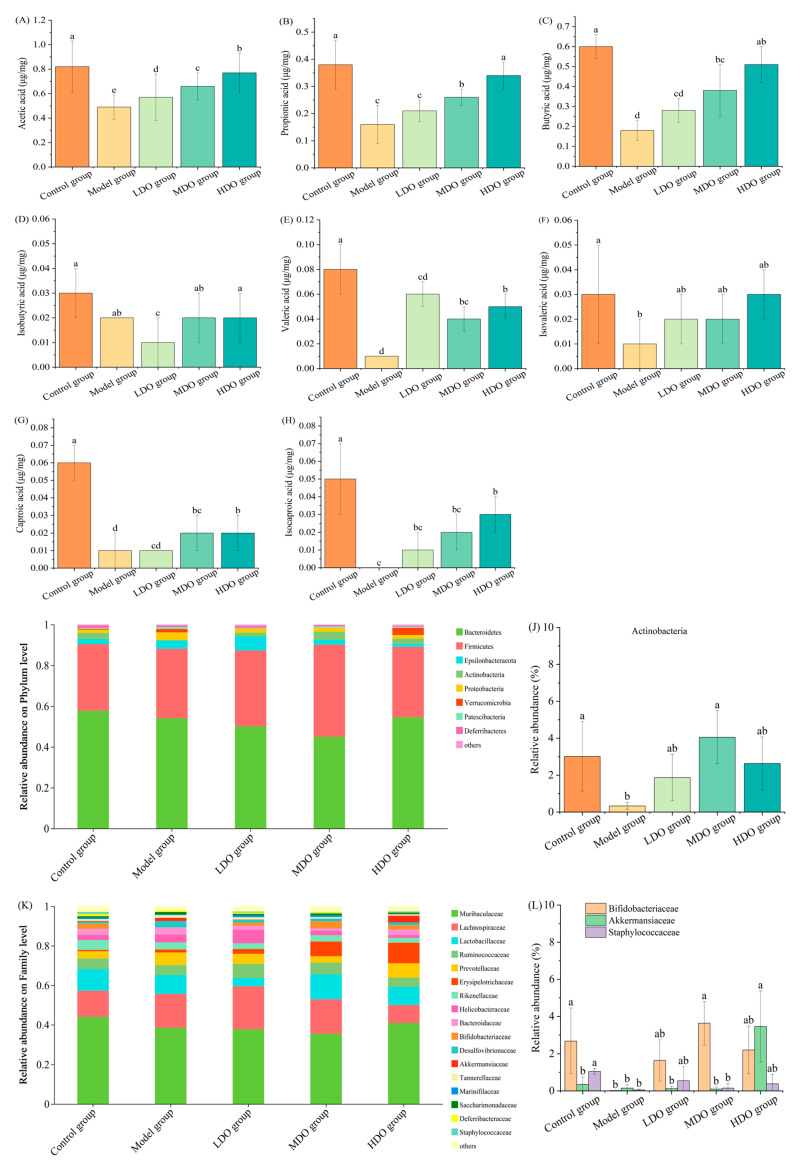

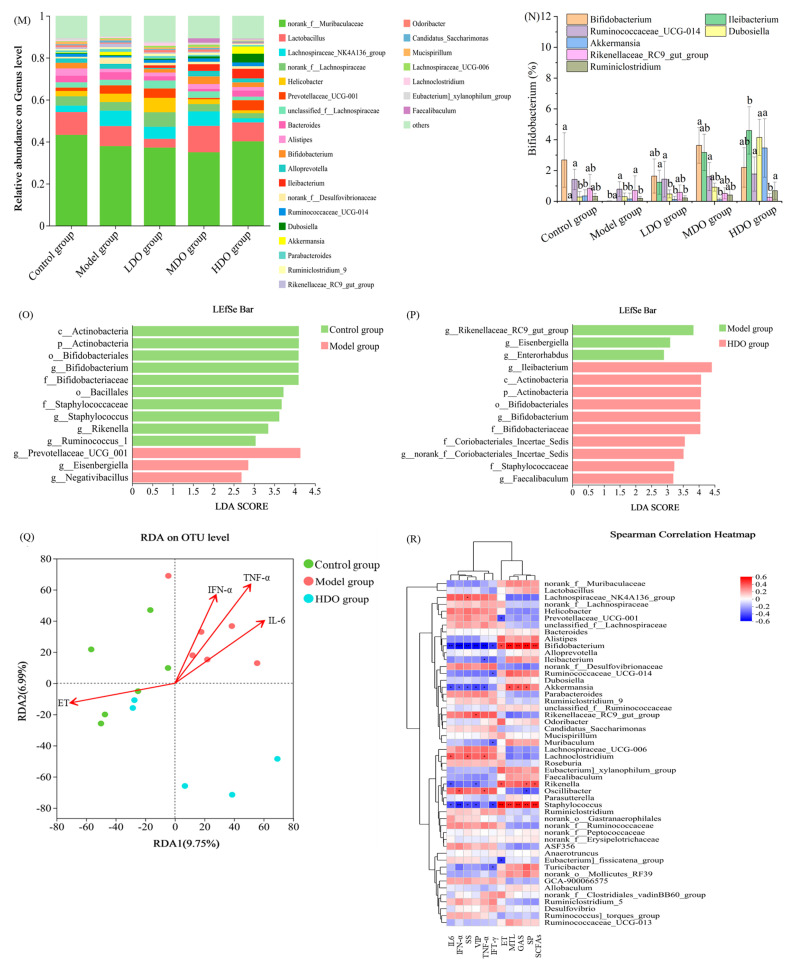
Effects of Chinese dwarf cherry kernel oil on short-chain fatty acids (SCFAs) in feces of different mouse groups (**A**–**H**). Relative abundances of the gut microbiota ((**I**,**J**) phylum level, (**K**,**L**) family level and (**M**,**N**) genus level). LDA diagrams: (**O**) between control group and model group; (**P**) between model group and HDO group). Redundancy analysis diagram (**Q**); Spearman correlation heatmap (**R**). (LDO group: low-dose oil group, MDO group: medium-dose oil group, HDO group: high-dose oil group). Note: The error bars represent the standard deviation (SD). Different lowercase letters indicate significant differences between different groups for the same indicator (*p* < 0.05). “*” indicates significant differences in the correlation between different physicochemical indicators and the same intestinal microflora (*p* < 0.05). “**” indicates highly significant differences in the correlation between different physicochemical indicators and the same intestinal microflora (*p* < 0.01).

SCFAs are also referred to as volatile fatty acids. These fatty acids exhibit diverse physiological actions, which include energy provision, the enhancement of intestinal circulation and the suppression of pathogenic microorganisms [20]. According to Figure 6A–H, a notable decline in the levels of most SCFAs was observed following constipation (*p* < 0.05). In contrast, mice in the MDO and HDO groups exhibited significantly elevated levels of acetic acid, propionic acid, butyric acid, valeric acid and caproic acid compared to the model group (*p* < 0.05). These findings indicate that CDC kernel oil can regulate SCFA concentrations, potentially mitigating constipation symptoms. Moreover, a higher dose of CDC kernel oil appeared to exhibit a significant effect on isocaproic acid (*p* < 0.05) compared to other oil groups.

### 3.6. Effects of CDC Kernel Oil on Gut Microbial Composition

Loperamide and CDC kernel oil seem to alter the microbial structure within the intestinal tract. The α-diversity of different mice groups were shown in Supplementary Materials (Table S3), as well as the result of partial least-squares discrimination analysis (PLS-DA, Figure S1). Furthermore, a comparison of the gut microbial composition across different groups was conducted at the phylum, family and genus levels, as illustrated in Figure 6I–N. At the phylum level (Figure 6I,J), Firmicutes and Bacteroidetes emerged as the dominant bacteria in the mouse feces, accounting for over 80% of the total bacteria, aligning with other studies [21,22]. The model group exhibited a decrease in the relative abundance of Actinobacteria (89%, *p* < 0.05), whereas the CDC kernel oil groups showed an increased proportion of Actinobacteria. At the family level (Figure 6K,L), *Muribaculaceae*, *Lachnospiraceae* and *Lactobacillaceae* were the primary bacteria present in the mouse feces. Compared with the control group, the model group exhibited significant reductions in the relative abundances of *Bifidobacteriaceae* and *Staphylococcaceae*, namely 92% and 95%, respectively (*p* < 0.05), while the relative abundance of *Erysipelotrichaceae* increased by 118% (*p* < 0.05). Furthermore, compared with the model group, the CDC kernel oil groups exhibited increased relative abundances of *Bifidobacteriaceae*, *Akkermansiaceae* and *Staphylococcaceae*. Notably, the HDO group showed a higher relative abundance of *Akkermansiaceae* than the other two oil treatment groups, while the MDO group displayed elevated levels of *Bifidobacteriaceae*, and the LDO group had a greater abundance of *Staphylococcaceae*. At the genus level (Figure 6M,N), the main bacteria in the feces of mice were *norank_f_Muribaculaceae*, *Lactobacillus*, *Lachnospiraceae_NK4A136_group* and *Helicobacter*. Compared with the control group, the model group exhibited reductions in the abundances of *Bifidobacteriaceae*, *Ruminococcaceae_UCG_014*, *Ruminococcaceae* and *Akkermansia*, along with an increase in *Desulfovibrionaceae*. Specifically, the relative abundance of *Bifidobacteriaceae* decreased by 99.25% (*p* < 0.05), *Ruminococcaceae_UCG_014* by 44.37%, *Ruminiclostridium* by 42.42% and *Akkermansia* by 57.14%, whereas that of *norank_f_Desulfovibrionaceae* increased by 288.46%. Following CDC kernel oil treatment, the relative abundances of *Bifidobacteriaceae*, *Ruminococcaceae_UCG_014*, *Ruminococcaceae* and *Akkermansia* were restored, while that of *norank_f_Desulfovibrionaceae* declined. Notably, the HDO group showed a significant difference compared to the model group (*p* < 0.05). Furthermore, the HDO group demonstrated a markedly higher *Akkermansia* abundance than the other four groups, reaching 9.89-fold that of the control group and 23.07-fold that of the model group. Moreover, after constipation was induced, *Lachnospiraceae_NK4A136_group*, *Bacteroides* and *Rikenellaceae_RC9_gut_group* were also increased (*p* < 0.05), and they were decreased after CDC kernel oil treatment (*p* < 0.05). Specifically, compared with the control and model groups, *Dubosiella* was checked out in the CDC kernel oil groups and exhibited a pronounced positive correlation with increasing doses of CDC kernel oil. A similar result was also observed for *Ileibacterium.*

We also employed linear discriminant analysis (LDA), accompanied by effect size measurement, to compare the taxonomic differences between the control and model groups (Figure 6O). Compared with the model group, *Bifidobacterium* and *Ruminococcaceae_1* were present in higher proportions in the control group; these are beneficial bacteria producing SCFAs. The different taxa between the model and HDO groups are shown in Figure 6P. *Ileibacterium* and *Bifidobacterium* were present in higher proportions in the HDO group compared with the LHI group.

### 3.7. Correlations Between Intestinal Microflora and Physicochemical Indices

Environmental factors may influence microbial community structures. Therefore, we investigated the possible association between the structure of the microbial community and the environmental variables (IL-6, TNF-α, IFN-α, IFN-γ, VIP, SS, ET, GAS, SP, MTL, SCFAs) through redundancy analysis (RDA). It showed that the two components could explain 16.74% of the total variation (Figure 6Q). The second component (RDA2) explained 6.99% of the variation. It was demonstrated that IL-6, TNF-α, IFN-α and ET were screened out as they showed a significant correlation with the intestinal microflora (*p* < 0.05). Furthermore, we conducted Spearman’s correlation analysis between the top 50 genera and the physicochemical indicators associated with constipation, as shown in Figure 6R. Interestingly, *Bifidobacterium* exhibited a positive relationship with SCFAs, ET, GAS, SP and MTL (*p* < 0.05), whereas it showed a negative correlation with IL-6, IFN-α, SS, VIP, TNF-α and IFT-γ (*p* < 0.05). *Akkermansia* displayed a positive correlation with SP, GAS and MTL (*p* < 0.05) but a negative one with TNF-α, IL-6, IFN-α, VIP and SS (*p* < 0.05). There was a significant positive correlation between *Rikenella* and SCFAs and SP, as well as ET (*p* < 0.05), and a negative one with VIP and IL-6 (*p* < 0.05). Additionally, *Ruminococcaceae_UCG_014* demonstrated a negative correlation with IFT-γ (*p* < 0.05).

## 4. Discussion

In this study, we investigated the therapeutic potential of CDC kernel oil in alleviating loperamide-induced constipation in mice. The oil’s rich nutritional profile (shown in Table S1 in Supplementary Materials)—characterized by high levels of squalene, unsaturated fatty acids (predominantly oleic and linoleic acids), sterols (notably β-sitosterol), minerals (especially Mg), vitamin E and γ-tocopherol—likely underpins its observed physiological effects, including laxative, anti-inflammatory and microbiota-modulating properties.

Over 80% of the oil is unsaturated, mainly consisting of monounsaturated (C18:1, MUFA) and polyunsaturated (C18:2, PUFA) fatty acids. Studies indicate that PUFA-rich diets enhance the gut microbiota’s diversity, contrasting with saturated fatty acid-rich diets [23], while MUFAs’ effects are less clear [24]. CDC kernel oil, rich in Mg, may modulate the gut microbiota, similarly to a seaweed ingredient that boosted microbial diversity in rats [25]. It also has higher selenium content (7.61 μg/100 g) than peanut and rapeseed oils, aiding immunomodulation and exerting anticancer and cardiovascular effects [26]. The vitamin E level in CDC kernel oil, primarily γ-tocopherol (44.84 mg/100 g), exceeds the levels in palm, sunflower and peanut oils [27]. While the antioxidant activity of tocopherols and tocotrienols generally follows the order of α > β > γ > δ, tocotrienols, especially γ-tocotrienol, show superior antioxidant effects in oils [28]. Squalene, with antioxidant, anticancer and anti-inflammatory properties, is traditionally sourced from deep-sea shark livers but can also be isolated from plants like CDC [29]. CDC kernel oil’s squalene content (19.69 mg/100 g) surpasses that of palm, soybean and sunflower oils [30]. The plant sterols in CDC kernel oil, particularly β-sitosterol (over 66.25% of total sterols), outperform those in rapeseed and peanut oils in lowering cholesterol and reducing inflammation [31,32]. Thus, CDC kernel oil is a rich source of bioactive compounds with antioxidant and anti-inflammatory benefits.

Consistent with prior reports [33], loperamide-induced constipation resulted in reduced fecal output, increased water absorption and delayed intestinal transit. These findings suggest that the constipation model was successfully constructed. CDC kernel oil notably increased the fecal count and wet weight, providing direct evidence of its laxative effects, while having no significant impact on fecal water content in mice. Additionally, it enhanced gastrointestinal transit and accelerated the first black defecation time, facilitating fecal discharge and indicating that CDC kernel oil promotes peristalsis to prevent constipation. Compared to mulberry and yellow tea extracts, which have been proven effective in alleviating constipation in mice [13,34], CDC kernel oil exhibited superior laxative effects. It matched the normal control group in the first black defecation time but surpassed it in the fecal count, weight and gastrointestinal transit rate. This may stem from CDC kernel oil-induced intestinal smooth muscle relaxation and its high magnesium ion content, likely in the form of magnesium sulfate—a potent laxative that distends the stomach and induces liquid stool [35]. Furthermore, oleic acid, the predominant fatty acid in CDC kernel oil, facilitates defecation, promotes intestinal villus and crypt development, enhances intestinal barrier function and boosts peptide YY levels, thereby influencing intestinal motility [36,37].

Significantly, we discovered that CDC kernel oil effectively normalized the MDA and NO levels post-constipation induction, enhanced GSH-PX and SOD enzymatic activity and boosted T-AOC. Several neurotransmitter receptors, potentially inhibiting colonic motility and causing slow transit in constipation, have been identified. MTL receptors, expressed in human duodenal and colonic intestinal neurons, have been developed into agonists and antagonists to treat gastrointestinal motility disorders. GAS, secreted mainly by the gastric sinus, duodenum and small intestinal mucosa, stimulates gastric acid secretion and promotes gastrointestinal motility. SS inhibits smooth muscle contraction and GAS secretion, while SP suppresses gastrointestinal mucosa secretion and stimulates intestinal movement. ET maintains vascular tension stability and supports the cardiovascular system, and VIP relaxes the gastrointestinal tract, regulating intestinal peristalsis [38]. Our study showed that constipation increased the serum SS and VIP levels while decreasing the MTL, GAS, ET and SP levels. CDC kernel oil treatment reversed these trends, suggesting that it improves colonic motility by balancing inhibitory (SS and VIP) and excitatory (MTL, GAS, ET and SP) neurotransmitters. Inflammatory mediators like TNF-α, IL-6, IFN-γ and IFN-α are key cytokines in inflammatory bowel disease. The loperamide-induced constipation model in mice significantly increased these mediators’ secretion, indicating intestinal damage. CDC kernel oil treatment effectively reduced these levels, confirming its anti-inflammatory immune response effect. Histopathological features are crucial in evaluating constipation symptoms and therapeutic effects [32]. Histological analysis revealed that constipation significantly reduced mucus cell numbers and thinned the inner intestinal wall. In the colon, the serous membrane, muscularis, submucosa, mucosa, crypts and goblet cells were damaged post-constipation induction. Our results demonstrate that CDC kernel oil protected both the small intestine and colon tissue.

Previous studies have reported a close link between bowel disease and the intestinal microbiota, with microbiota regulation demonstrating a significant disease-modifying effect [39,40]. Our findings revealed that the primary microbial communities in our mouse model were Firmicutes and Bacteroidetes at the phylum level; *Muribaculaceae*, *Lachnospiraceae* and *Lactobacillaceae* at the family level; and *norank_f_Muribaculaceae*, *Lactobacillus*, *Lachnospiraceae_NK4A136_group* and *Helicobacter* at the genus level, consistent with the results from similar mice models in other studies [13,34]. Neither loperamide nor CDC kernel oil treatment affected the richness and diversity of the main fecal microbial communities. However, constipation induction significantly reduced the Actinobacteria phylum, *Bifidobacteriaceae* and *Staphylococcaceae* families and *Bifidobacterium*, *Ruminococcaceae_UGG_014* and *Rumnimiclostridium* genera. Conversely, the CDC kernel oil groups exhibited a significant increase in these taxa, along with the *Akkermansia* genus. *Bifidobacteriaceae*, which are important beneficial autochthonous bacteria in the human gastrointestinal microbiota, play a crucial role in maintaining gastrointestinal health and preventing constipation and gastroenteritis. As common probiotics, they produce acetic acid, lactic acid and other short-chain fatty acids (SCFAs) to stimulate peristalsis and inhibit intestinal bacterial fermentation. *Ruminococcaceae*, primarily inhabiting the cecum and colon, degrade diverse polysaccharides and fibers, producing SCFAs that inhibit harmful bacterial growth [41]. *Akkermansia*, the most abundant mucus-soluble bacteria in the healthy gut, are confirmed to be beneficial in humans. Notably, our study observed an increase in *Akkermansia* in the HDO group, possibly due to CDC kernel oil promoting goblet cell differentiation and mucus production, creating a favorable environment for *Akkermansia* growth [42]. Additionally, *Dubosiella* and *Ileibacterium* showed a positive correlation with increasing oil doses in the CDC kernel oil groups. In mouse models, Dubosiella’s intestinal abundance is linked to obesity, aging and Alzheimer’s. Research shows that *Dubosiella* outperforms *Akkermansia* in repairing colonic barriers and easing inflammation caused by dextran sulfate sodium, indicating its probiotic potential. It is also more effective than resveratrol in reducing oxidative stress, improving vascular function and redistributing gut microbiota components like *Ileibacterium*. *Dubosiella* is positively tied to butyric acid levels and negatively to IL-1β, IL-6 and TNF-α mRNA, as well as positively to IL-10. *Ileibacterium* has functions similar to those of *Dubosiella* [43,44,45,46]. SCFAs, which are the end products of microbial fermentation in the mammalian colon, are related to constipation. Our study demonstrated that CDC kernel oil increased the SCFA concentrations to prevent constipation, aligning with the changes in gut microbiota diversity and taxonomic composition. The SCFA types varied with the CDC kernel oil dosage, with the HDO group containing the most types, including acetic, propionic, butyric, valeric, hexanoic and isovaleric acids. Previous studies have reported that SCFAs may promote water and electrolyte absorption, enhance epithelial cell proliferation, influence gastrointestinal motility, increase mesenteric blood flow and exert other physiological effects. Changes in the fecal water content, gastrointestinal transit rate and intestinal histology were consistent with SCFA variations. To further investigate the gut microbiota associated with neurotransmitter receptors, inflammatory mediators and SCFAs at the OUT level, we employed RDA analysis and Spearman’s correlations. After administering CDC kernel oil to constipated mice, we observed a positive correlation between SCFA and excitatory neurotransmitter levels and *Bifidobacterium*, while inhibitory neurotransmitter and inflammatory mediator levels were negatively correlated with *Bifidobacterium*. *Akkermansia* and *Ruminococcaceae_UCG_014* were linked to certain neurotransmitters and inflammatory mediators.

These results suggest that CDC kernel oil regulates immunity and maintains intestinal homeostasis, potentially stimulating immune system activation. The therapeutic effects of CDC kernel oil may stem from synergistic interactions among its constituents. Mg likely enhanced motility via osmotic mechanisms; unsaturated fatty acids and vitamin E contributed to anti-inflammatory and antioxidant effects; and squalene and sterols may have supported microbial growth and mucosal repair. While our findings highlight CDC kernel oil as a promising functional food for constipation management, the precise mechanisms linking its compounds to microbial and host pathways warrant further investigation. Future studies should explore dose–response relationships and long-term safety to validate its translational potential.

## 5. Conclusions

CDC kernel oil appeared to relieve constipation induced by loperamide in mice, as evaluated by the first black fecal defecation time, fecal number, fecal wet weight, water content of feces and gastrointestinal transit rate. CDC kernel oil reduced inhibitory neurotransmitters and increased excitability neurotransmitters, two anti-oxidases’ activity and the fecal SCFA content. The histological morphology showed that mucus cells in the intestinal tract were improved by CDC kernel oil treatment. Meanwhile, CDC kernel oil also increased the abundance of some beneficial bacteria, including *Bifidobacterium*, *Akkernansia*, *Ruminococcaceae_UGG_014* and *Ruminiclostridium*, and decreased *norank_f _Desulfovibrionaceae*, which is correlated with gastrointestinal diseases. In addition, it was identified that the gut microbiota was associated with receptors of neurotransmitters, mediators of inflammation and SCFAs. The findings provide a theoretical foundation for the use of CDC kernel oil as a promising functional food in the prevention of constipation. In addition, future studies are needed to address some unexplained phenomena and problems. Firstly, it is crucial to identify the primary constituents of CDC kernel oil that possess effective constipation-relieving properties. Subsequently, an investigation into the mechanisms governing the regulation of constipation by the intestinal microbiota is necessary. Nonetheless, as an initial investigative effort, our study verifies the beneficial application of CDC kernel oil to prevent constipation.

## Figures and Tables

**Table 1:** Effects of Cerasus humilis kernel oil on colon tissue of mice.

Group	Small Intestinal Villus Length (μm)	Colon Crypt Depth (μm)	Colon Pathology Score
Control group	1142.54 ± 101.01 ^a^	333.95 ± 15.55 ^a^	1.00 ± 0.70 ^e^
Model group	507.90 ± 57.66 ^e^	132.41 ± 9.85 ^d^	25.00 ± 0.70 ^a^
LDO group	677.07 ± 56.22 ^d^	161.04 ± 4.87 ^c^	15.40 ± 0.89 ^b^
MDO group	870.21 ± 33.22 ^c^	176.65 ± 12.84 ^c^	7.60 ± 0.54 ^c^
HDO group	984.72 ± 35.03 ^b^	259.01 ± 16.46 ^b^	2.60 ± 0.54 ^d^

Note: Different lowercase letters indicate significant differences between different groups for the same indicator (*p* < 0.05).

## Data Availability

Data are contained within the article.

## Supplementary Materials

The following supporting information can be downloaded at https://www.mdpi.com/article/10.3390/nu18020319/s1: Table S1. Nutrient composition of CDC kernel oil. Table S2. Body mass and food intake of KM mice during the observation period in acute toxicity test. Table S3. α-diversity estimators of different mice groups. Figure S1. Partial least squares discriminant analysis (PLS-DA) of gut microbiota in different mice groups (LDO group: Low dose oil group, MDO group: Medium dose oil group, HDO: High dose oil group).

## Nomenclature

**Abbreviation**	**Definition**
LDO	Low-dose oil
MDO	Medium-dose oil
HDO	High-dose oil
CDC	Chinese dwarf cherry
bw	Body weight
NO	Nitric oxide
MDA	Malondialdehyde
GSH-PX	Glutathione peroxidase
SOD	Superoxide dismutase
MTL	Motilin
T-AOC	Total antioxidative capacity
GAS	Gastrin
SS	Somatostatin
ET	Endothelin
SP	Substance P
VIP	Vasoactive intestinal peptide
IL-6	Interleukin-6
INF-α	Interferon-α
INF-γ	Interferon-γ
TNF-α	Tumor necrosis factor-α
SCFAs	Short-chain fatty acids
ICP-MS	Inductively coupled plasma mass spectrometry
RP-HPLC	Reversed-phase high-performance liquid chromatography
GC-MS	Gas chromatography–mass spectrometry
LDA	Linear discriminant analysis
RDA	Redundancy analysis
IBD	Inflammatory bowel disease
ARRIVE	Animal Research: Reporting In Vivo Experiments
ELISA	Enzyme-linked immunosorbent assay
OTU	Operational taxonomic unit
PLS-DA	Partial least-squares discrimination analysis

## References

[1] Kojima R., Doihara H., Nozawa K., Kawabata-Shoda E., Yokoyama T., Ito H. (2009). Characterization of Two Models of Drug-Induced Constipation in Mice and Evaluation of Mustard Oil in These Models. Pharmacology.

[2] Cirillo C., Capasso R. (2015). Constipation and Botanical Medicines: An Overview. Phytother. Res..

[3] Rao S.S.C., Brenner D.M. (2021). Efficacy and Safety of Over-the-Counter Therapies for Chronic Constipation: An Updated Systematic Review. Am. J. Gastroenterol..

[4] Turnbaugh P.J., Baeckhed F., Fulton L., Gordon J.I. (2008). Diet-Induced Obesity Is Linked to Marked but Reversible Alterations in the Mouse Distal Gut Microbiome. Cell Host Microbe.

[5] Quigley E.M.M., Spiller R.C. (2016). Constipation and the Microbiome: Lumen versus Mucosa!. Gastroenterology.

[6] Johanson J.F., Kralstein J. (2007). Chronic Constipation: A Survey of the Patient Perspective. Aliment. Pharmacol. Ther..

[7] Wang X., Yin J. (2015). Complementary and Alternative Therapies for Chronic Constipation. Evid. Based Complement. Altern. Med..

[8] Saneian H., Tavakkol K., Adhamian P., Gholamrezaei A. (2013). Comparison of Lactobacillus Sporogenes plus Mineral Oil and Mineral Oil Alone in the Treatment of Childhood Functional Constipation. J. Res. Med. Sci..

[9] Chau C.-F., Wu S.-H. (2006). The Development of Regulations of Chinese Herbal Medicines for Both Medicinal and Food Uses. Trends Food Sci. Technol..

[10] Mu X.P., Aryal N., Du J.M., Du J.J. (2015). Oil Content and Fatty Acid Composition of the Kernels of 31 Genotypes of Chinese Dwarf Cherry [*Cerasus humilis* (Bge.) Sok.]. J. Hortic. Sci. Biotechnol..

[11] Piironen V., Toivo J., Lampi A.M. (2002). Plant sterols in cereals and cereal products. Cereal Chem..

[12] Jia Z.-F., Wang J.-L., Pan W., Hu J. (2024). *Croton tiglium* L. Seeds Ameliorate Loperamide-Induced Constipation via Regulating Gastrointestinal Hormones and Gut Microbiota before and after Processing. J. Ethnopharmacol..

[13] Cao P.Q., Li X.P., Ou Yang J., Jiang R.G., Huang F.F., Wen B.B., Zhang X.N., Huang J.A., Liu Z.H. (2021). The Protective Effects of Yellow Tea Extract Against Loperamide-Induced Constipation in Mice. Food Funct..

[14] Lv H., Niu J., Pan W., Wang Y., Wang L., Wang M., Shi Y., Zhang G., Al Hamyari B., Wang S. (2024). Stool-Softening Effect and Action Mechanism of Free Anthraquinones Extracted from *Rheum palmatum* L. on Water Deficit-Induced Constipation in Rats. J. Ethnopharmacol..

[15] Zhang J., Zhao Y., Ren D., Yang X. (2020). Effect of Okra Fruit Powder Supplementation on Metabolic Syndrome and Gut Microbiota Diversity in High Fat Diet-Induced Obese Mice. Food Res. Int..

[16] Lin Q., Liu M., Erhunmwunsee F., Li B., Mou Y., Wang S., Zhang G., Tian J. (2022). Chinese Patent Medicine Shouhui Tongbian Capsule Attenuated Loperamide-Induced Constipation through Modulating the Gut Microbiota in Rat. J. Ethnopharmacol..

[17] Choi Y.J., Park J.W., Kim J.E., Lee S.J., Gong J.E., Jung Y.-S., Seo S., Hwang D.Y., Choi Y.J., Park J.W. (2021). Novel Characterization of Constipation Phenotypes in ICR Mice Orally Administrated with Polystyrene Microplastics. Int. J. Mol. Sci..

[18] Zhu R., Wang Y., Zhang L., Guo Q. (2012). Oxidative Stress and Liver Disease. Hepatol. Res..

[19] Han X.Y., Xu Z.R., Wang Y.Z., Huang Q.C. (2006). Effect of Cadmium on Lipid Peroxidation and Activities of Antioxidant Enzymes in Growing Pigs. Biol. Trace Elem. Res..

[20] Yu H., Guo Z., Shen S., Shan W. (2016). Effects of Taurine on Gut Microbiota and Metabolism in Mice. Amino Acids.

[21] Turnbaugh P.J., Hamady M., Yatsunenko T., Cantarel B.L., Duncan A., Ley R.E., Sogin M.L., Jones W.J., Roe B.A., Affourtit J.P. (2009). A Core Gut Microbiome in Obese and Lean Twins. Nature.

[22] Xu P., Wang J., Hong F., Wang S., Jin X., Xue T., Jia L., Zhai Y. (2017). Melatonin Prevents Obesity Through Modulation of Gut Microbiota in Mice. J. Pineal Res..

[23] Patterson E., O’ Doherty R.M., Murphy E.F., Wall R., O’ Sullivan O., Nilaweera K., Fitzgerald G.F., Cotter P.D., Ross R.P., Stanton C. (2014). Impact of Dietary Fatty Acids on Metabolic Activity and Host Intestinal Microbiota Composition in C57BL/6J Mice. Br. J. Nutr..

[24] Ye Z., Xu Y.-J., Liu Y. (2021). Influences of Dietary Oils and Fats, and the Accompanied Minor Content of Components on the Gut Microbiota and Gut Inflammation: A Review. Trends Food Sci. Technol..

[25] Crowley E.K., Long-Smith C.M., Murphy A., Patterson E., Murphy K., O’Gorman D.M., Stanton C., Nolan Y.M., Crowley E.K., Long-Smith C.M. (2018). Dietary Supplementation with a Magnesium-Rich Marine Mineral Blend Enhances the Diversity of Gastrointestinal Microbiota. Mar. Drugs.

[26] Hamilton S.J. (2004). Review of Selenium Toxicity in the Aquatic Food Chain. Sci. Total Environ..

[27] Ershow A.G., Wong-Chen K. (1990). Chinese Food Composition Tables An Annotated Translation of the 1981 Edition Published by the Institute of Nutrition and Food Hygiene, Chinese Academy of Preventive Medicine, Beijing. J. Food Compos. Anal..

[28] Gustinelli G., Eliasson L., Svelander C., Alminger M., Ahrné L. (2018). Supercritical CO_2_ Extraction of Bilberry (*Vaccinium myrtillus* L.) Seed Oil: Fatty Acid Composition and Antioxidant Activity. J. Supercrit. Fluids.

[29] Sheng Y.Y., Xiang J., Wang K.R., Li Z.Y., Li K., Lu J.L., Ye J.H., Liang Y.R., Zheng X.Q. (2022). Extraction of Squalene from Tea Leaves (*Camellia sinensis*) and Its Variations with Leaf Maturity and Tea Cultivar. Front. Nutr..

[30] Dongsheng Z., Yalin X., Qingzhe J., Xingguo W., Dong Z., Cheng Z. (2013). Determination of Squalene in Oil-Tea Camellia Seed Oil. China Oils Fats.

[31] Cofán M., Ros E. (2015). Clinical Application of Plant Sterol and Stanol Products. J. AOAC Int..

[32] Lee H.-Y., Kim J.-H., Jeung H.-W., Lee C.-U., Kim D.-S., Li B., Lee G.-H., Sung M.-S., Ha K.-C., Back H.-I. (2012). Effects of Ficus Carica Paste on Loperamide-Induced Constipation in Rats. Food Chem. Toxicol..

[33] Wang L., Hu L., Yan S., Jiang T., Fang S., Wang G., Zhao J., Zhang H., Chen W. (2017). Effects of Different Oligosaccharides at Various Dosages on the Composition of Gut Microbiota and Short-Chain Fatty Acids in Mice with Constipation. Food Funct..

[34] Chen Y., Li Q., Zou Y., Zhou Z.X., Feng W.W., Bao Y.T., Ma R.H., Ji P.C., Wu J., Yang L.Q. (2014). Protective Effect of Mulberry Extract against Pb-Induced Learning and Memory Deficits in Mice. Biomed. Environ. Sci. BES.

[35] Ali R., Irfan M., Akram U., Vaince M., Hassan K., Maqsood A., Aslam A., Amaan N., Qamar A., Memon S. (2021). Efficacy of Natural Formulation Containing Activated Charcoal, Calcium Sennosides, Peppermint Oil, Fennel Oil, Rhubarb Extract, and Purified Sulfur (Nucarb^®^) in Relieving Constipation. Cureus.

[36] Compher C.W., Kinosian B.P., Rubesin S.E., Ratcliffe S.J., Metz D.C. (2009). Energy Absorption Is Reduced With Oleic Acid Supplements in Human Short Bowel Syndrome. J. Parenter. Enter. Nutr..

[37] Fang Z., Chen Y., Li Y., Sun L., Deng Q., Wang J., Gooneratne R., Fang Z., Chen Y., Li Y. (2022). Oleic Acid Facilitates Cd Excretion by Increasing the Abundance of Burkholderia in Cd-Exposed Mice. Int. J. Mol. Sci..

[38] Jiang H., Dong J., Jiang S., Liang Q., Zhang Y., Liu Z., Ma C., Wang J., Kang W. (2020). Effect of Durio Zibethinus Rind Polysaccharide on Functional Constipation and Intestinal Microbiota in Rats. Food Res. Int..

[39] Deng Y., Li M., Mei L., Cong L.M., Liu Y., Zhang B.B., He C.Y., Zheng P.Y., Yuan J.L. (2018). Manipulation of Intestinal Dysbiosis by a Bacterial Mixture Ameliorates Loperamide-Induced Constipation in Rats. Benef. Microbes.

[40] Qin J., Li R., Raes J., Arumugam M., Burgdorf K.S., Manichanh C., Nielsen T., Pons N., Levenez F., Yamada T. (2010). A Human Gut Microbial Gene Catalogue Established by Metagenomic Sequencing. Nature.

[41] Shang Q., Shan X., Cai C., Hao J., Li G., Yu G. (2016). Dietary Fucoidan Modulates the Gut Microbiota in Mice by Increasing the Abundance of *Lactobacillus* and *Ruminococcaceae*. Food Funct..

[42] Guo C., Cui Q., Cheng J., Chen J., Zhao Z., Guo R., Dai X., Wei Z., Li W. (2021). Probiotic-Fermented Chinese Dwarf Cherry [*Cerasus humilis* (Bge.) Sok.] Juice Modulates the Intestinal Mucosal Barrier and Increases The Abundance of Akkermansia in the Gut in Association with Polyphenols. J. Funct. Foods.

[43] Zhang Y., Tu S., Ji X., Wu J., Meng J., Gao J., Shao X., Shi S., Wang G., Qiu J. (2024). *Dubosiella newyorkensis* Modulates Immune Tolerance in Colitis via the L-Lysine-Activated AhR-IDO1-Kyn Pathway. Nat. Commun..

[44] Liu T., Wang J., Zhang C., Zhao L., Sheng Y., Tao G., Xue Y. (2023). Gut Microbial Characteristical Comparison Reveals Potential Anti-Aging Function of *Dubosiella newyorkensis* in Mice. Front. Endocrinol..

[45] Chen Y., Ye S., Shi J., Wang H., Deng G., Wang G., Wang S., Yuan Q., Yang L., Mou T. (2024). Functional Evaluation of Pure Natural Edible Ferment: Protective Function on Ulcerative Colitis. Front. Microbiol..

[46] Wan F., Han H., Zhong R., Wang M., Tang S., Zhang S., Hou F., Yi B., Zhang H. (2021). Dihydroquercetin Supplement Alleviates Colonic Inflammation Potentially through Improved Gut Microbiota Community in Mice. Food Funct..

