# Supplementary material for: The Effects of Chinese Dwarf Cherry (Cerasus humilis) Kernel Oil on Defecation and the Gut Microbiota in Constipated Mice
Source: Nutrients. 2026 Jan 19;18(2):319. doi: 10.3390/nu18020319 (PMC12844711; doi:10.3390/nu18020319)
Supplement: Supplementary file 1 [file nutrients-18-00319-s001.zip › nutrients-4050477-supplementary.pdf]

The effect of Chinese dwarf cherry (*Cerasus humilis*) kernel oil on defecation and gut microbiota in constipated mice

Jingyu Gao [a,b], Yumin Dai [a,b], Zhe Liang [a,b], Nan Chen [a,b], Xilong Li [a,b], Xin Wen [a,b], Yuanying Ni [a,b], Mo Li [a,b*]

[a] College of Food Science and Nutritional Engineering, China Agricultural University, No. 17 Qinghua East Road, Beijing 100083, China; B20223060512@cau.edu.cn (J.G.); 17801035462@163.com (Y.D.); liangzhe@cau.edu.cn (Z.L.); 18801086980@163.com (N.C.); LiXiLong2026@outlook.com (X.L.); xin.wen@cau.edu.cn (X.W.); nyy@cau.edu.cn (Y.N.)

[b] National Engineering Research Center for Fruits and Vegetables Processing, No. 17 Qinghua East Road, Beijing 100083, China

*Correspondence: limo0125@cau.edu.cn; Tel./Fax: +86-10-62737514

1. Methods

1.1 Nutrient analysis of CDC kernels oil

Mineral element concentrations of CDC kernels oil were determined using inductively coupled plasma-mass spectrometry (ICP-MS, GB 5009.268-2016). Vitamin E contents were determined using reversed-phase high-performance liquid chromatography (RP-HPLC, GB 5009. 82-2016). The standard curves of α-tocopheryl, β-tocopheryl, γ-tocopheryl and δ-tocopheryl were Y=4.368X-0.117 (R^2=0.9999), Y=6.061X+0.025 (R^2=1.0000), Y=5.452X-0.134 (R^2=0.9999) and Y=4.791X-0.079 (R^2=0.9999) respectively. The composition of fatty acids was analyzed utilizing gas chromatography–mass spectrometry (GC-MS, GB 5009.168-2016, External Standard

Method). Sterol compositions were determined by GC-MS (Piironen et al., 2002). The standard curve for Clerosterol was $Y=3.710\times10^{-3}X-1.200\times10^{3}$ ($R^2=0.9998$). The standard curve for Campesterol was $Y=1.754\times10^{-3}X-6.940\times10^{3}$ ($R^2=0.9997$). The standard curve for Stigmasterol was $Y=1.124\times10^{-3}X-3.002\times10^{3}$ ($R^2=0.9997$). The standard curve for β-sitosterol was $Y=3.124\times10^{-3}X+1.600\times10^{3}$ ($R^2=0.9998$). The standard curve for Delta7-Avenasterol was $Y=2.107\times10^{-3}X+3.580\times10^{3}$ ($R^2=0.9999$). Squalene content was determined by GC-MS (SN/T 4785-2017). The standard curve for squalene was $Y= 1.439\times10^{3}X-1.986\times10^{3}$ ($R^2=0.9990$).

1.2 The acute oral toxicity analysis of CDC kernel oil

In order to better evaluate the safety of CDC kernel oil, the toxicological analysis was carried out using acute toxicity test. Throughout the study, the different groups were assigned to different cages. Thirty-six 6-week-old SPF (Specific Pathogen Free) Kunming mice (27 ± 1 g) were divided randomly into 6 groups (6 mice per group). Before administration, the mice were fasted for 12-16 hours. Then, the mice received the same volume (0.1mL/10 g mice weight) of CDC kernel oil with different concentration (0, 1125, 2250, 4500, 9000 and 15000 mg/kg) by gavage. The toxicity and death of mice were observed for 7 days. Based on the above preliminary results, forty 6-week-old SPF Kunming mice were assigned to two groups: a control group and a CDC kernel oil group, with an equal distribution of 20 mice in each. After fasting for 12-16 hours, the mice received 15 g/kg·body weight (bw) of CDC kernel oil by gavage. The toxicity and death of mice were observed for 14 days.

2. Results

2.1 Chemical Analysis

After the green technology of supercritical carbon dioxide fluid extraction, the extraction rate of Chinese dwarf cherry kernel oil (CDC kernel oil) was 28.52±0.51%. The nutrient composition of CDC kernel oil was shown in Table S1. These results indicated that CDC kernel oil contained beneficial nutrient contributing to human health. In addition, amygdalin was not detected in CDC kernel oil.

Table S1. Nutrient composition of CDC kernel oil

Nutrient composition	Content (/100g)
Squalene	19.69±0.68 mg
Fatty acid	
Palmitic acid	4.38±0.12 g
Palmitoleic acid	0.42±0.01 g
Stearic acid	0.82±0.01 g
Oleic acid	58.00±0.67 g
Linoleic acid	31.10±0.49 g
Linolenic acid	0.12±0.00 g
Sterols	
Clerosterol	12.93±0.14 mg
Campesterol	13.09±0.12 mg
Stigmasterol	2.68±0.04 mg
β-Sitosterol	108.95±1.03 mg
Oats sterol	26.81±0.15 mg
Mineral element	
Fe	1.3 mg
Zn	0.18 mg
Mg	41.69 mg
Na	3.49 mg
K	0.79 mg
Ca	6.52 mg
P	0.80 mg
Se	7.61 µg
Vitamin E	55.24 mg
α- tocopheryl	4.82 mg
γ- tocopheryl	44.84 mg
δ-tocopheryl	5.58 mg

2.2 The acute oral toxicity of CDC kernel oil

The maximum dose of 15 g/kg·bw of CDC kernel oil was used to gavage mice during the observation period, all the mice survived and did not show symptoms of poisoning, so it can be preliminarily determined that CDC kernel oil is non-toxic. As shown in Table S2, there was no significant difference in body weight and food intake between the mice and the control group ($p < 0.05$), and the behavior and activity of all mice were normal. After the experiment, all mice were dissected and no abnormalities were observed in the main organs. Therefore, the LD50 of CDC kernel oil is greater than 15 g/kg. According to the national standard GB 15193.3.2014 "Acute Oral Toxicity Test", it can be determined that CDC kernel oil is actually non-toxic.

Table S2. Body mass and food intake of KM mice during the observation period in acute toxicity test

Day	Body bass		Food intake	
	**control group**	**CDC oil group**	**control group**	**CDC oil group**
1	23.60±1.05^{a}	24.15±0.98^{a}	5.95±0.24^{a}	6.15±0.28^{a}
4	24.75±1.71^{a}	25.54±1.27^{a}	6.28±0.18^{a}	6.54±0.27^{a}
7	26.77±1.06^{a}	27.58±1.19^{a}	6.59±0.21^{a}	6.69±0.30^{a}
10	26.77±1.06^{a}	30.61±1.44^{a}	7.24±0.18^{a}	7.28±0.28^{a}
14	32.27±1.71^{a}	32.44±1.76^{a}	8.28±0.12^{a}	8.29±0.23^{a}

Data were expressed as mean ± standard deviation. The same small letter represents no significant differences between control group and CDC oil group ($p > 0.05$).

2.3 Effect of CDC kernel oil on the diversity of fecal microbiota

To assess the impact of CDC kernel oil on gut microbiota, we employed metagenomic α-diversity analysis targeting the V_3-V_4 region of the 16S rRNA gene sequences (shown in Table S3). This analysis involved examining alpha diversity metrics at the OUT level, encompassing indices for community richness (specifically, chao and ace), indices for community diversity (Shannon and simpson), as well as the index for community coverage. Table S3 revealed that neither community richness, diversity, nor coverage

underwent significant alterations across all treatment groups, both before and after constipation induction. Furthermore, partial least-squares discrimination analysis (PLS-DA) conducted at the OUT level depicted the overall microbial composition differences in Fig. S1. After constipation was induced the model group shifted to the right side of Fig. S1 compared to the control group, suggesting a modification in gut microbiota composition due to loperamide. Treatment with CDC kernel oil distinctly separated the gut microbiome profiles of the LDO and MDO groups from both the control and model groups, indicating a substantial shift in fecal microbiota structure. In contrast, the microbiome profile of HDO still had overlap with the model group.

Table S3. α-diversity estimators of different mice groups

Groups	Community diversity		Community richness		Community coverage
	shannon	simpson	ace	chao	coverage
Control group	4.4088±0.2529	0.0260±0.0058	520.50±30.19	525.40±37.06	0.9974±0.0004
Model group	4.1887±0.1520	0.0331±0.0065	497.04±53.95	497.60±49.62	0.9975±0.0004
LDO group	4.4156±0.1635	0.0281±0.0060	504.70±22.63	507.43±24.16	0.9977±0.0001
MDO group	4.1912±0.2912	0.0352±0.0151	489.80±22.75	487.64±28.60	0.9975±0.0004
HDO group	4.1351±0.2662	0.0376±0.0148	500.33±41.48	518.03±38.19	0.9973±0.0003

Data were expressed as mean ± standard deviation.

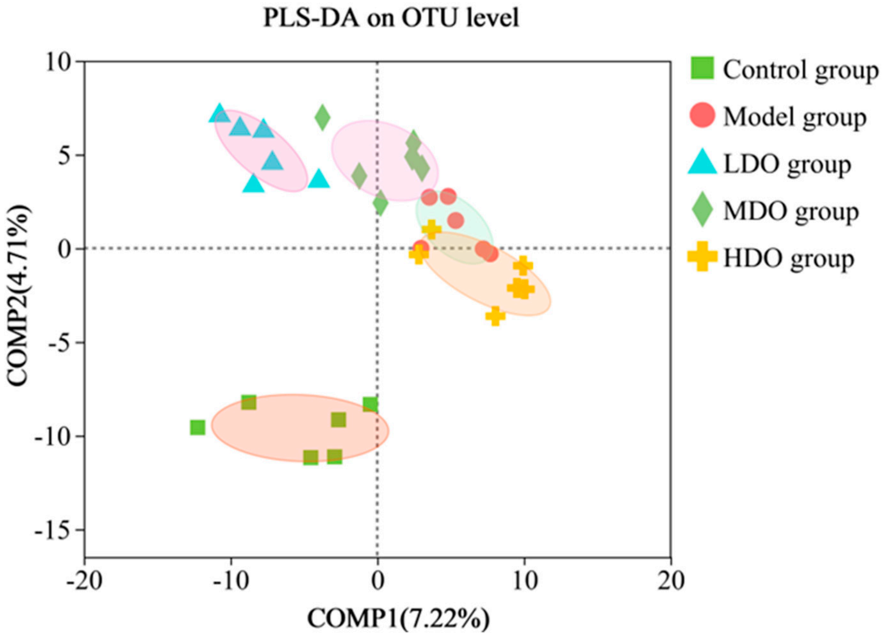

Figure S1. Partial least squares discriminant analysis (PLS-DA) of gut microbiota in different mice groups (LDO group: Low dose oil group, MDO group: Medium dose oil group, HDO: High dose oil group).

References

Piironen, V., Toivo, J., & Lampi, A. M. (2002). Plant sterols in cereals and cereal products. *Cereal Chemistry*, *79*(1), 148–154. https://doi.org/10.1094/CCHEM.2002.79.1.148
